# Drug-Eluting Stents: Their Preventative/Prophylactic Role Against Gemcitabine Induced Acute Coronary Syndrome

**DOI:** 10.7759/cureus.16384

**Published:** 2021-07-14

**Authors:** Jaafar A Hamdan, Kerolos N Youssef, Aafreen Khan, Mohammed A Abdalla, Christine M Zakhary, Hiam Rushdi, Safeera Khan

**Affiliations:** 1 Medicine, American University of Antigua, St. John, ATG; 2 Internal Medicine, California Institute of Behavioral Neurosciences & Psychology, Fairfield, USA; 3 Psychiatry, California Institute of Behavioral Neurosciences & Psychology, Fairfield, USA

**Keywords:** acute coronary syndrome, coronary artery vasospasm, interventional cardiology, primary percutaneous coronary intervention (pci), drug eluting stents, chemotherapy-related toxicity, gemcitabine, 5 fluorouracil, capecitabine

## Abstract

Acute coronary syndrome (ACS), a subdivision of ischemic cardiac disease, is the sudden occlusion of coronary vessels that results in decreased blood supply to heart muscles and possible infarction. Though some of the etiologies are hypertension, hyperlipidemia, diabetes mellitus, and tobacco; certain types of chemotherapies play a major role. Percutaneous coronary intervention (PCI) has shown lifesaving results via drug-eluting stent (DES) deployment into occluded vessels. In this study, DES utilization among patients receiving chemotherapy will be assessed to observe if it provides any prevention against ACS.

Articles were systematically screened in three databases such as PubMed, PubMed Central (PMC), and Medical Literature Analysis and Retrieval System Online (MEDLINE) using keywords and Medical Subject Heading (MeSH) terms for applicable articles. Additionally, a few relevant articles from the Cochrane Library, Molecular Diversity Preservation International (MDPI), and The New England Journal of Medicine were also used. Inclusion/exclusion criteria were applied post article screening via title and abstracts. Quality appraisal check was done using the Scale for the Assessment of Narrative Review Articles (SANRA) checklist, A Measurement Tool to Assess Systematic Reviews (AMSTAR) checklist, Cochrane bias assessment tool, and Joanna Briggs Institute (JBI) checklist. Ten related studies were strictly reviewed. DES did not appear to play a preventable role against ACS during chemotherapy as no study was found assessing DES prophylactically and its efficacy in cancer patients.

Future clinical trials on DES prophylactic use might be beneficial to evaluate if ACS adversities of chemotherapy can be prevented. This review is of significant benefit as cardiovascular adversities would not impede chemotherapy efficacy as cardiac adversities would not be part of the equation.

## Introduction and background

According to the World Health Organization (WHO), approximately 3.7 million people worldwide died due to acute coronary syndrome (ACS) in 2012, indicating that cardiovascular disease remains the leading cause of death globally [1]. ACS, a subdivision of ischemic heart disease, is classically induced by fatty plaques, depositing in the coronary vessel walls, ultimately hindering the delivery of oxygen and nutrients to the heart. Some risk factors for ACS are aging, hypertension, hypercholesterolemia, diabetes, tobacco, obesity, and COVID-19 infection. 

From a pathophysiologic perspective, atheroma - a plaque consisting of macrophage cells/debris containing fat, calcium, and connective tissues - is the main factor in ACS induction [2,3]. It creates an engorged arterial region by tightening the vessel lumen and impeding adequate blood flow. ACS is classically caused by plaque rupture that exposes thrombogenic material, thereby activating the coagulation cascade. This forms a thrombus, resulting in muscle infarction. The severity of the myocardial infarction (MI) damage depends on the time to reperfusion and injury induced by ischemia-reperfusion (I/R), which leads to a flow of cellular and humoral reaction [4]. Some complications occurring with ACS are arrhythmias, acute decompensated heart failure, cardiogenic shock, ventricular wall rupture, and papillary muscle-induced murmurs.

ACS can be clinically acknowledged via certain symptoms depending on gender, age group, and comorbidities. Typical symptoms include angina, pain radiation to upper extremities, upper abdomen and neck/jaw, nausea/vomiting, dyspnea, and diaphoresis. Some diagnostic measures are electrocardiography, biomarkers evaluation for myonecrosis such as Troponin I, Troponin T, and Creatine kinase-MB (CK-MB). When confirmed, it's imperative to get the patient to a catheter lab within ninety minutes for a percutaneous coronary intervention (PCI) with drug-eluting or bare-metal stents.

PCI is used worldwide. Drug-eluting stent (DES) however, is overall superior to bare-metal-stents as re-stenosis occurs at a lesser rate [5]. It is possibly due to the longer duration of dual antiplatelet therapy use in DES versus bare-metal-stent, as re-epithelization takes longer, ultimately decreasing the chance of re-stenosis and ACS. DES consists of a metal mesh, a polymer encasing the metal mesh, and an antiproliferative drug [1,6]. PCI works via balloon catheter insertion through the femoral or radial artery under X-ray imaging, which is then inflated in the stenosed region to permit blood flow. And DES, for example, is then implanted to ensure vessel patency [1]. In addition, DES contains antiproliferative agents (e.g., paclitaxel, sirolimus), possibly preventing neointimal growth and re-stenosis [1]. However, DES still has adversities; polymer coating has been linked with thrombotic events of the stent [7]. Overall, adverse events associated with PCI include coronary artery complications (e.g., perforation, distal embolization, stent thrombosis), MI, stroke, or death [8,9,10].

Though coronary artery disease (CAD) has avenues of treatments, it does retain complications with certain comorbidities such as cancer; an important consideration to account for since it's the second leading cause of death globally, according to the CDC. Chemotherapeutic agents used for malignancies have unfavorable cardiac effects and vascular toxicity. Chemotherapy-induced cardiovascular complications are often due to endothelial dysfunction with vasodilation and anti-inflammatory factors impairment [11]. A point of note especially as most chemotherapeutic agents further enhance platelet activity via a decrease of nitric oxide (NO), which is exacerbated through the normal procoagulant mechanism seen in cancer [11,12]. Therefore, the presence of CAD is a potential complication for those undergoing chemotherapy as certain cytokines/chemokines such as growth factors in several cancers lead to thrombus events, which in turn lead to ACS [13].

Antimetabolites (e.g., gemcitabine, capecitabine, 5-fluorouracil) chemotherapy are assessed in this review. 5- Fluorouracil (5-FU), for instance, works via inhibiting thymidylate synthase inhibitor, which prevents the synthesis of pyrimidine thymidylate for DNA replication. Angina, arrhythmias, ventricular tachycardia, MI, and dose-dependent increase in red blood cell viscosity are some of the related adversities [11,12]. Vasospasm associated with 5-FU causes vascular damage via reducing endothelial NO synthase activity and endothelium-independent vasoconstriction via protein kinase C, leading to prinzmetal-type angina [11,12]. Gemcitabine is a potent inhibitor of DNA synthesis; inside the cell, gemcitabine is phosphorylated to gemcitabine monophosphate via deoxycytidine kinase, then converted to gemcitabine di- and triphosphate (active metabolites) [8]. In deduction, cardiovascular complications are common and, interestingly enough, have increased in analogs with cancer survival [13]. 

An exploratory knowledge gap exists in determining how PCI can decrease the likelihood of ACS during chemotherapy treatment. According to a review, PCI has been performed in patients dissatisfied with quality of life due to symptoms related to ischemic heart disease or caused by adverse events due to medical treatment [14]. In essence, there is minute to no known knowledge of using DES prophylactically, perhaps before undergoing chemotherapy. Its study would be of significance in the possible prevention of ACS caused by vasospastic episodes that are in turn induced by certain chemotherapeutic adversities, and would greatly increase patient treatment compliance and survival. Our systematic review will analyze if DES has a preventable/prophylactic role in patients undergoing chemotherapy, gemcitabine in this example, to prevent ACS.

## Review

Protocol

This systematic review was conducted using the Preferred Reporting Items for Systematic Reviews and Meta-Analyses (PRISMA) guidelines [15].

Inclusion/exclusion criteria 

The literature search was done to isolate studies that demarcate the preventative role of DES in ACS in patients aged 45 or above undergoing chemotherapy. Inclusion criteria were human studies aged 45 or greater, published in English from January 1, 2006 to April 1, 2021, as full-text papers. We included randomized controlled trials, reviews, systematic reviews, clinical trials, meta-analyses, and case-control studies. Studies done on animal species, pediatric population, patients under 45, or written in other languages were excluded.

Data source and strategy 

The research was conducted using PubMed, PubMed Central (PMC), Medical Literature Analysis and Retrieval System Online (MEDLINE), Multidisciplinary Digital Publishing Institute (MDPI), Cochrane Library, and The New England Journal of Medicine (NEJM). Research in the database PubMed was conducted on April 24, 2021. The search for applicable articles was done using relative concepts ("Acute coronary syndrome," "Cytarabine," and "Drug-eluting stents"). It was then complemented with keywords via the boolean term "OR," displayed in Table 1 after the use of some Medical Subject Headings (MeSH) such as "preventative," "treatment," "Pathology," "therapeutic use," "pharmacology," and "adverse effects."

**Table 1:** PubMed Search Builders The keywords were combined using the boolean term "OR" and combined with their respective search builder obtained from PubMed using MeSH terms.

Concepts	Keywords	PubMed Search Builder
Acute coronary syndrome	Acute coronary syndrome, Coronary artery plaque rupture, Heart attack, Myocardial infarction, coronary artery clot formation, STEMI	( "Acute Coronary Syndrome/chemically induced"[Majr] OR "Acute Coronary Syndrome/complications"[Majr] OR "Acute Coronary Syndrome/drug therapy"[Majr] OR "Acute Coronary Syndrome/prevention and control"[Majr] OR "Acute Coronary Syndrome/therapy"[Majr] )
Cytarabine/Gemcitabine	Chemotherapy, Antimetabolite, Gemcitabine, Fludarabine, Pemetrexed, Fludarabine, Capecitabine, Cytarabine	( "Cytarabine/administration and dosage"[Majr] OR "Cytarabine/adverse effects"[Majr] OR "Cytarabine/physiology"[Majr] OR "Cytarabine/therapeutic use"[Majr] OR "Cytarabine/toxicity"[Majr] )
Drug-eluting Stents	Drug-eluting stents, Paclitaxel-eluting stents, Everolimus-eluting stents, Percutaneous coronary intervention	( "Drug-Eluting Stents/adverse effects"[Majr] OR "Drug-Eluting Stents/pharmacology"[Majr] OR "Drug-Eluting Stents/therapeutic use"[Majr] )

In addition, restrictions to MeSH major topic were applied. Finally, all concepts and keywords were combined into an algorithm using the boolean term "AND" to include relevant articles, as shown in Table 2.

**Table 2:** Comprehensive Full MeSH strategy Algorithm The PubMed search builder algorithms were combined into one comprehensive algorithm using the boolean term "AND", applicable articles.

Full MeSH Strategy: Combining Individual PubMed Search Builders	Articles
Acute Coronary syndrome OR Coronary artery Plaque rupture OR Heart attack OR Myocardial Infarction OR coronary artery clot formation OR STEMI OR ( "Acute Coronary Syndrome/chemically induced"[Majr] OR "Acute Coronary Syndrome/complications"[Majr] OR "Acute Coronary Syndrome/drug therapy"[Majr] OR "Acute Coronary Syndrome/prevention and control"[Majr] OR "Acute Coronary Syndrome/therapy"[Majr] ) AND Chemotherapy OR Antimetabolite OR Gemcitabine OR Fludarabine OR Pemetrexed OR Fludarabine OR Capecitabine OR ( "Cytarabine/administration and dosage"[Majr] OR "Cytarabine/adverse effects"[Majr] OR "Cytarabine/physiology"[Majr] OR "Cytarabine/therapeutic use"[Majr] OR "Cytarabine/toxicity"[Majr] ) AND Drug Eluting Stents OR Paclitaxel-eluting stents OR Everolimus-eluting stents OR Percutaneous Coronary Intervention OR ( "Drug-Eluting Stents/adverse effects"[Majr] OR "Drug-Eluting Stents/pharmacology"[Majr] OR "Drug-Eluting Stents/therapeutic use"[Majr] )	The algorithm used for research had a yield of 72, 750 Articles

Data extraction

Articles were screened according to titles, abstracts, and full-text features via two independent reviewers, JH and KN, to fully rule in/out relevant articles. The items extracted from each study included study design, year of publication, age range, and outcome. Studies gathered by one reviewer were analyzed by other reviewers for precision and worthiness. In case of dissidence, divergences were resolved by a mutual discussion on the study in question.

Bias evaluation tools

The quality appraisal was done using the following tools portrayed in Table 3:

**Table 3:** Quality Appraisal Tools The following tools were used for article appraisal for each type of respective article. Only articles satisfying >70% of the checklist quality parameters were included in the systematic review.

Quality appraisal tools	Articles
Cochrane Risk Bias Assessment Tool	Randomized Controlled Trials
Scale for the Assessment of Narrative Review Articles (SANRA) Checklist	Research paper w/out methods section
Assessing the Methodological Quality of Systematic Reviews (AMSTAR) Checklist	Systematic reviews
Joanna Briggs Institute (JBI) Checklist	Case reports

Results 

Study Identification and Selection

**Figure 1:**
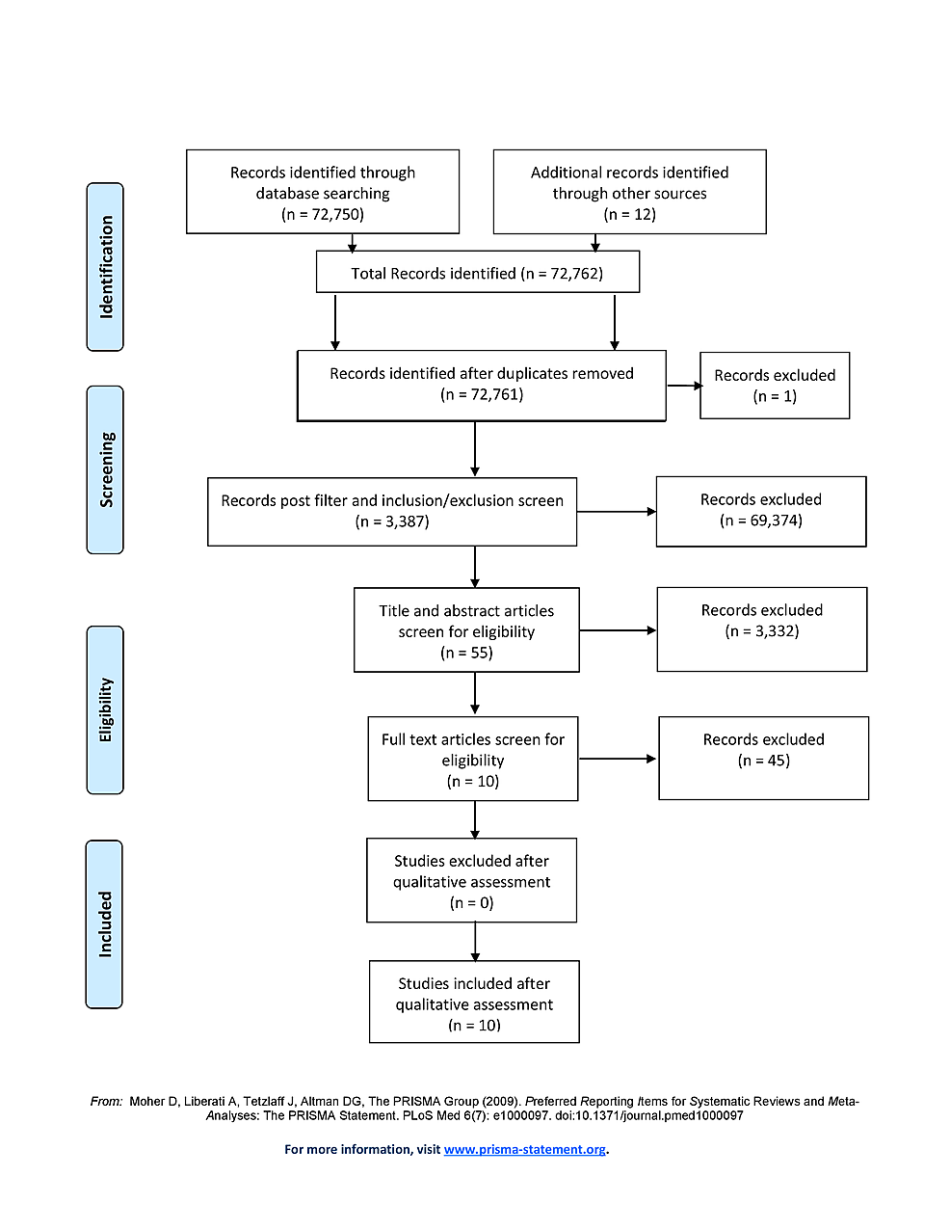
PRISMA Flow Diagram PRISMA: Preferred Reporting Items for Systematic Reviews and Meta-Analyses. Flow diagram displaying the screening and selection process of articles obtained from PubMed and other Databases post inclusion/exclusion criteria application.

There were six databases used for relevant articles: PubMed, PMC, MEDLINE, MDPI, Cochrane Library, and NEJM. The research generated 72,762 articles: post duplicates removal, 72,761 articles were applied to inclusion/exclusion criteria, resulting in 3,387 articles. Articles were screened using title relevance, abstracts, and full texts, which yielded 10 articles that met quality assessment. Figure 1 illustrates the search process in the shape of a PRISMA flow diagram.

Quality Assessment 

Quality assessment was done for 10 articles using various tools: SANRA checklist (n= five), AMSTAR checklist (n= two), COCHRANE bias assessment tool (n= one), and JBI checklist (n= two). All 10 articles were applied in this systematic review as all satisfied the cut-off (>70%). 

Findings of Studies 

Based on studies conducted, it was observed, patients undergoing chemotherapy experience ACS/vasospastic complications regardless of existing cardiac comorbidities [16-19]. In total, when compiling studies used in this review, 983 patients in the past experienced vascular complications post-chemotherapy treatment, including gemcitabine, for this study purpose [16-19]. However, no literature studies were done on DES prophylactic application prior to chemotherapy treatment to prevent ACS or related vascular complications [16-19]. Secondly, certain patients with pre-existing DES placements did experience stent re-stenosis/vascular complications post chemotherapy treatments [16,18,19]. In inference, DES in those studies lacked efficacy in the prevention of vascular adversities.

Discussion

As ACS has been associated with certain risk factors such as hypertension, hyperlipidemia, and diabetes mellitus, there has been a significant role of chemotherapy-induced ACS and other vasospastic complications [16-19]. Therefore, while drug-eluting stents are used in the ACS population, this systematic review will be utilized to understand if DES has a prophylactic preventative role against ACS in patients undergoing chemotherapy treatments. 

Chemotherapy pharmacology 

Chemotherapy is a pharmacological treatment used to treat a variety of malignancies. It can be used as a sole treatment or post-surgical intervention, referred to as adjunctive treatment. When chemotherapy is used to shrink the tumor, other treatments like radiation or surgery can also be used as neoadjuvant treatment. Moreover, chemotherapy may be utilized to alleviate symptoms induced by malignancy, called palliative chemotherapy treatment. Some examples of chemotherapies are capecitabine, 5-fluorouracil, fludarabine, and gemcitabine.

Chemotherapy, such as gemcitabine, originally investigated as an antiviral agent, was developed into a chemotherapy drug based on its positive conclusive in in vitro and in vivo anti-tumor activity [13]. This, furthermore, was strengthened by the evidence that gemcitabine had an anti-growth factor against solid and hematological cancer cell lines, indicated as a single agent in the treatment of patients with metastatic pancreatic cancer [13,20]. Other malignancies where gemcitabine is used are head and neck cancer, mesothelioma, and ovarian cancer [13]. However, in certain cancers, such as non-small cell lung cancer, it is often used in conjunction with other chemotherapies [21]. 

Similar to certain chemotherapies such as cytarabine, gemcitabine is a prodrug, necessitating the uptake by the cell for intracellular phosphorylation via the enzyme Deoxycytidine kinase (dCK) [13]. This converts gemcitabine into gemcitabine monophosphate, becoming gemcitabine di- and triphosphate, which are the active metabolites [13]. Unlike cytarabine, gemcitabine can have several targets intracellularly; inhibitory action mainly on DNA synthesis [22-24].

**Figure 2:**
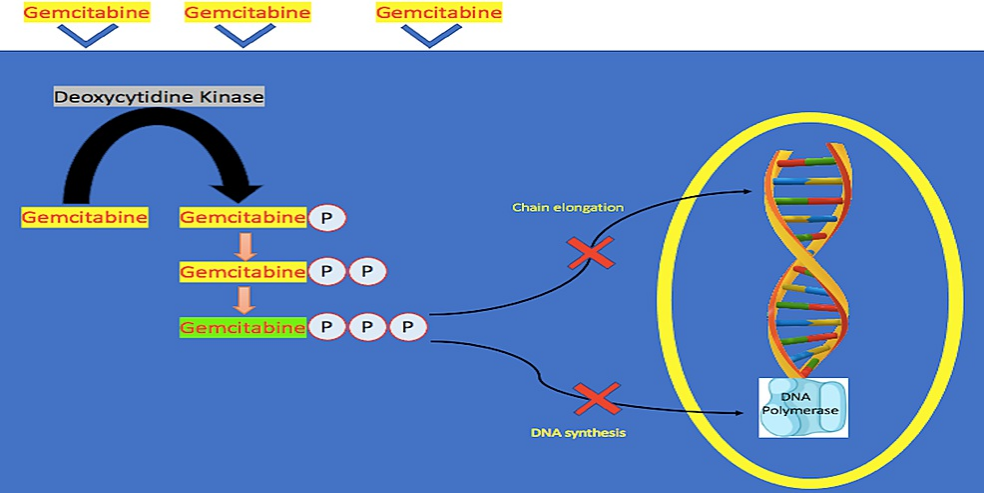
Gemcitabine Conversion To Active Form Depiction of gemcitabine binding onto the nucleoside transporters of the tumorous cells. The drug agent is activated through a series of enzymatic intracellular phosphorylation via deoxycytidine kinase. This ultimately leads to the death of the tumor cells via the inhibition of DNA polymerase and the elongation synthetic process of DNA.

Gemcitabine interferes with cancer DNA synthesis by inhibiting DNA polymerase via the active metabolites of gemcitabine (gemcitabine triphosphate) and incorporation into DNA [24,25]. This ultimately creates a termination of the chain elongation synthesis process [25]. Secondly, the non-terminal positioning of gemcitabine triphosphate in the DNA chains inhibits the detection and restoration of DNA errors via the DNA repair enzymes [26,27]. This finally leads to apoptosis of the cancerous cell [13]. Figure 2 illustrates this complex pharmacokinetic/pharmacodynamic process.

Though gemcitabine presents an assuring mechanism in its efficacy, an obstacle to consider is sensitivity/resistance, which is determined by the presence of certain nucleoside transporters acting as gates that take up the drug [28]. Hence, it is concluded that in the event of cells being deficient in those transporters, the tumor overall is resistant to that particular pharmaceutical agent [28].

In conclusion, gemcitabine has obtained a crucial character in treating a variety of solid tumors [13]. Moreover, due to its strong nature of lacking cross-resistance with other anti-tumor agents (e.g., cisplatin, taxanes, and anthracyclines), it is an effective drug for combination chemotherapy treatments in certain malignancies [13].

Chemotherapy-induced cardiovascular adversities**

Chemotherapy treatment, apart from many side-effects, presents a series of cardiotoxicity impacts on patients, limiting their clinical use in certain patient populations due to certain comorbidities, particularly those with a history of pre-existing cardiac disease. Moreover, many of the chemotherapeutic agents interact with cardiovascular signaling, presenting crucial lethal adverse outcomes, predominantly during increased cardiac demand during physical exertion [29]. Among the several types of chemotherapy agents and their respective unfavorable outcomes, antimetabolite agents will be discussed.

Pyrimidine analogs are antimetabolites (e.g., capecitabine, gemcitabine, cytarabine fluorouracil, and fludarabine), carrying significant clinical considerations to be evaluated such as coronary vasospastic and thromboembolic ischemic events [29]. Coronary artery disease has been a solid additive factor in antimetabolites-induced coronary vasospastic complications [29]. However, there have been patients with no existing cardiac disease who experienced vasospastic episodes during treatment with this drug class during the first few days of treatment [29]. Though vasospastic incidents are mere side-effects of these chemotherapeutic agents, they can pose insidious complications or even death in certain clinical settings. As the coronary vessels experience vasospasm (e.g., prinzmetal angina), it can induce arrhythmias that interfere with the native electrical conductive pathway of the heart. As a result, the heart muscle won't effectively nor adequately pump/perfuse the peripheral organs, including its muscular structure. 

This can lead to a vicious cycle, ultimately leading to a possible acute heart failure, resulting in organs, including the heart, not being supplied with adequate oxygen and nutrients. A possible outcome of acute heart failure is pulmonary edema, which can interfere with blood oxygenation and ultimately lead to muscular heart infarctions.

However, in a patient with pre-existing coronary artery disease, these side effects present a disastrous outcome as any compromise in blood supply can lead to possible ACS or even death. The reason being, in the setting of atherosclerotic coronary vessels, any blood supply compromise will likely cause serious complications as poor blood supply to the heart muscles is already in existence. Hence, lethal outcomes such as arrhythmias and myocardial infarction can ensue.

Additionally, another point to assess in patients undergoing chemotherapy treatments is their coagulative state. Patients with cancer tend to be in a hypercoagulable state; chemotherapeutic agents, among other adversities, can further exacerbate this hypercoagulable state. This, combined with the setting of atherosclerosis disease in coronary vessels, can be a possibility as to why/how vasospastic episodes occur due to chemotherapeutic agents that can lead to complications such as myocardial infarction. 

Drug-eluting stents preventative role in chemotherapy-induced ACS

PCI treatment has been used and displayed a significant positive outcome in efficacy intervention in acute myocardial infarction. A metal catheter is utilized to guide and deploy DES or bare-metal stent to alleviate the occlusion present in the coronary artery and maintain its patency. In this systematic review, the main focus will be on the study of drug-eluting stents. DES, in addition, will be evaluated from a cardio-oncology perspective, concluding if they hold a preventative role against ACS in patients undergoing chemotherapy treatment through potential prophylactic use. The following case studies were evaluated:

A case report published by Ozturk features a 59-year-old female with risk factors for CAD and coronary stenosis assessed on prior coronary angiography [16]. The patient had metastatic leiomyosarcoma and developed chest pain induced via vasospasm and acute left bundle branch block post gemcitabine infusion [16]. This was worsened due to pre-existing CAD [16]. The patient was treated with antianginal therapy and PCI and discharged soon after with no further cardiac complications. The patient, however, died five months later due to disease progression [16]. Though the most common cardiotoxic effect is coronary ischemia due to antimetabolites [30], DES was not shown to have a preventative/protective role in preventing ACS in patients undergoing chemotherapy, specifically gemcitabine. In conclusion, gemcitabine-induced coronary vasospasm combined with existing CAD was the cause of ACS [16]. Similar to 5-FU, the gemcitabine mechanism of coronary ischemia is not well-covered, but coronary vasospasm is a possible mechanism [16]. Furthermore, other culprits of ischemia include endothelial dysfunction and coronary thrombosis [16]. DES has not been shown or been experimented with to check for any preventative/prophylactic role against ACS induced via chemotherapy. 

An article published by Hilmi et al. assessed the adversities of gemcitabine treatment - cardiovascular complications including MI, pericardial diseases, supraventricular arrhythmias, and heart failure [17]. Gemcitabine is utilized either as a monotherapy or in combination to treat various malignancies of the lung, pancreas, bladder, breast, ovary, bile duct carcinomas, lymphomas, and uterine sarcomas [31]. Cardiotoxicities induced via gemcitabine have been reported in clinical trials. A review of 979 patients treated by gemcitabine in 22 phase-2 trials displayed the incidence of MI, heart failures, arrhythmias, and pericarditis to be 0.5%, 0.4%, 0.2%, and 0.1%, respectively [32]. 

A case report by Camaro et al. on a 61-year-old male with a history of ACS and metastatic colorectal cancer [18]. The patient underwent PCI due to acute ST-elevation myocardial infarction (STEMI) post capecitabine therapy [18]. Though no stenosis was detected on PCI, the patient was stated to have been administered capecitabine (oral 5-FU analog) a day prior, which is known to induce coronary vasospasm [18]. The patient had EKG abnormalities (early repolarization, ST elevation in inferolateral leads, and peaked T waves) [18]. The PCI showed no changes from a previous one done three years prior [18]. However, the chest pain and EKG were resolved with calcium channel blockers and discontinued capecitabine post-consultation [18]. In patients with metastatic colorectal cancer, capecitabine is a treatment that has replaced IV 5-FU, which has a toxic consequence on coronary endothelium and myocardium, which can result in MI, ventricular arrhythmias, and sudden death [33]. In conclusion, there was no positive, beneficial role of DES against ACS prevention in chemotherapy patients.

A case series report published by Lee and Yoon evaluated two patients [19]:

The first, 56-year-old male patient with a history of hypertension and unstable angina had total occlusion of the left circumflex artery (LCX) stent, which resulted in a PCI for proximal anterior descending artery and LCX artery [19]. The angiographic intervention was done, and the patient was discharged [19]. The patient returned after one month for neoadjuvant chemotherapy (paclitaxel+ carboplatin) for lung squamous cell carcinoma [19]. Post ten minutes of paclitaxel infusion, the patient developed diaphoresis, dyspnea, chest pain, and hypotension [19]. Patient EKG showed ST-segment elevation of 4 mm in V2-V4 for which abciximab administration, aspiration thrombectomy, and balloon angioplasty was performed, and the patient recovered [19].

A second patient, a 68-year-old male with a history of pancreatic adenocarcinoma and a known history of hypertension and three-vessel CAD, was treated 13 years prior via PCI [19]. The patient was mechanically intubated post-surgery with elevation in cardiac enzymes accompanied by muscular motion abnormalities in the right coronary artery territory and left ventricular dysfunction [19]. PCI was done due to significant occlusion of the left anterior descending (LAD) artery, which resulted in ventricular fibrillation with pulseless ventricular tachycardia 20 minutes after the procedure [19]. On further intervention, total occlusion of the proximal LAD stent with thrombus was found [19]. The patient recovered but died later due to cancer progression [19]. Thrombosis occurred both in the stented vessel region and the untreated native coronary artery segment [19]. Cancer is a major risk factor for hypercoagulation conditions (e.g., deep vein thrombosis, pulmonary embolism, and arterial thromboembolism) [34,35]. It is believed that tumors produce procoagulant factors, decrease inhibition factors toward coagulation, inflammatory cytokines, and impaired fibrinolysis as contributors to the pro-coagulative state [36]. When treatment is implemented, such as chemotherapy, the pro-coagulative state is amplified further [20]. Though the main mechanism is not fully understood of the ACS, it has been projected to be due to vasospasm of coronary vessels or endothelial dysfunction [37,38]. In conclusion, the vascular stents in place did not seem to play a preventable role against vascular complications (e.g., ACS).

Limitations

PCI with DES implantation has been used clinically to treat ACS and treat clinically symptomatic blocked coronary vessels. Although this systematic review did not find a preventative/prophylactic role of DES against ACS in patients undergoing chemotherapy treatment, there were certain limitations in this study. The study included only papers published in English on patients aged 45 and above, from January 1, 2006 to April 1, 2021. This systematic review study could have excluded clinical trials/case reports published in different languages on patients with an age that violates the inclusion criteria or perhaps animal studies that could have studied clinical usage of DES in a prophylactic manner. Moreover, a limitation to this hypothesis could have been met with an ethical dilemma; non-maleficence, as PCI therapy does have unfavorable adversities such as stent re-stenosis.

## Conclusions

This study was conducted to understand if DES plays a unique clinical role by being implanted in coronary arteries of patients bound for chemotherapy treatment to avoid cardiac complications (e.g., infarctions). Antimetabolite class of chemotherapy works ultimately via DNA synthesis inhibition upon its activation through a series of intracellular phosphorylation. Though antimetabolites such as gemcitabine have acquired a unique favorable role by lacking cross-resistance with other classes of chemotherapy, making it a strong candidate in combination treatment with other drug classes, it still carries serious clinical risks. Concerning the cardiovascular system, patients occasionally experience acute chest pain, coronary vasospastic episodes, which can lead to thrombus formation in the healthy population, stent restenosis in patients with a history of PCI treatment, and in rare cases, MI. Hence, it was concluded that DES did not have a preventative function apropos ACS in those undergoing chemotherapy as no literature in this review has portrayed as such. A future recommendation in cardio-oncology is conducting clinical trials in prophylactically implanting DES in patients ahead of their chemotherapy and comparing results with a control group to assess efficacy in preventing ACS.
